# Gallic Acid Alleviates Visceral Pain and Depression via Inhibition of P2X7 Receptor

**DOI:** 10.3390/ijms23116159

**Published:** 2022-05-31

**Authors:** Lequan Wen, Lirui Tang, Mingming Zhang, Congrui Wang, Shujuan Li, Yuqing Wen, Hongcheng Tu, Haokun Tian, Jingyi Wei, Peiwen Liang, Changsen Yang, Guodong Li, Yun Gao

**Affiliations:** 1 Joint Program of Nanchang University and Queen Mary University of London, Nanchang University, 461 Bayi Avenue, Nanchang 330006, China; jp4217118145@qmul.ac.uk (L.W.); jp4217118218@qmul.ac.uk (L.T.); jp4217118147@qmul.ac.uk (H.T.); jp4217119235@qmul.ac.uk (C.Y.); 2 Department of Physiology, Basic Medical College, Nanchang University, 461 Bayi Avenue, Nanchang 330006, China; zhangmm1117@126.com (M.Z.); wenyuqing0211@163.com (Y.W.); gc77li@163.com (G.L.); 3 Second Clinic Medical College, Nanchang University, 461 Bayi Avenue, Nanchang 330006, China; ncusuqyzxwcr@163.com (C.W.); zololo116@163.com (S.L.); lpwhafffe@163.com (P.L.); 4 Basic Medical College, Nanchang University, 461 Bayi Avenue, Nanchang 330006, China; 18322950360@wo.cn (H.T.); v2899001289@163.com (J.W.); 5 Jiangxi Provincial Key Laboratory of Autonomic Nervous Function and Disease, 461 Bayi Avenue, Nanchang 330006, China

**Keywords:** gallic acid, P2X7 receptor, visceral pain, depression, hippocampus, spinal cord, dorsal root ganglion

## Abstract

Chronic visceral pain can occur in many disorders, the most common of which is irritable bowel syndrome (IBS). Moreover, depression is a frequent comorbidity of chronic visceral pain. The P2X7 receptor is crucial in inflammatory processes and is closely connected to developing pain and depression. Gallic acid, a phenolic acid that can be extracted from traditional Chinese medicine, has been demonstrated to be anti-inflammatory and anti-depressive. In this study, we investigated whether gallic acid could alleviate comorbid visceral pain and depression by reducing the expression of the P2X7 receptor. To this end, the pain thresholds of rats with comorbid visceral pain and depression were gauged using the abdominal withdraw reflex score, whereas the depression level of each rat was quantified using the sucrose preference test, the forced swimming test, and the open field test. The expressions of the P2X7 receptor in the hippocampus, spinal cord, and dorsal root ganglion (DRG) were assessed by Western blotting and quantitative real-time PCR. Furthermore, the distributions of the P2X7 receptor and glial fibrillary acidic protein (GFAP) in the hippocampus and DRG were investigated in immunofluorescent experiments. The expressions of *p*-ERK1/2 and ERK1/2 were determined using Western blotting. The enzyme-linked immunosorbent assay was utilized to measure the concentrations of IL-1β, TNF-α, and IL-10 in the serum. Our results demonstrate that gallic acid was able to alleviate both pain and depression in the rats under study. Gallic acid also reduced the expressions of the P2X7 receptor and *p*-ERK1/2 in the hippocampi, spinal cords, and DRGs of these rats. Moreover, gallic acid treatment decreased the serum concentrations of IL-1β and TNF-α, while raising IL-10 levels in these rats. Thus, gallic acid may be an effective novel candidate for the treatment of comorbid visceral pain and depression by inhibiting the expressions of the P2X7 receptor in the hippocampus, spinal cord, and DRG.

## 1. Introduction

Irritable bowel syndrome (IBS) is a gastrointestinal disorder that is characterized by altered defecation habits, abdominal discomfort, and abdominal pain. It is said that IBS affects the lives of 10–15% of the global population [1]. Since IBS is the most prevalent functional gastrointestinal disorder (FGID) in chronic visceral pain [2,3,4,5], we selected it as the investigatory target in this study representing chronic visceral pain. The severity of IBS symptoms varies from person to person, from enervating to mild [6]. Moreover, in IBS patients, long-term neuroplastic changes have occurred in the brain–gut axis, which results in chronic abdominal pain [7,8]. Visceral hyperalgesia may be related not only to peripheral mechanisms within the intestinal wall but also to increased neurotransmitters released in the spinal cord and brain [9]. Aside from the nociceptive symptoms, IBS is commonly accompanied by other intestinal or non-intestinal comorbidities, 20–60% of which involve anxiety or depression [2]. Both mucosal inflammation and neuroinflammation are involved in the pathophysiology of IBS [10], which might be responsible for the comorbidity development of visceral pain and depression. In this study, we constructed the comorbidity of visceral pain and depression of IBS models for research purposes and tried to identify a common effective target for the comorbid visceral pain and depression.

To date, increasing evidence suggests that purinergic receptors are strongly related to visceral hyperalgesia; commonly studied ones are the P2X1, P2X3, P2X2/3, P2X7, P2Y1, and P2Y2 receptors [11,12,13,14,15,16]. The P2X7 receptor, which is the central factor in the process of inflammation [17], is found to have an enhanced effect in visceral hyperalgesia [15,18]. Furthermore, Antonio et al. observed that the inhibition of the P2X7 receptor expression at the nerve terminals with oxidized ATP could suppress inflammation pain [19]. Additionally, Jarvis proved that the P2X7 receptors in microglia participate in neuropathic pain [20]. It was also suggested that the P2X7 receptors could regulate the production of IL-1β, thus inducing inflammation and neuropathic pain [21,22]. Coincidentally, there is growing evidence showing that the P2X7 receptor is a crucial player in depression. Various studies have demonstrated the central role of the P2X7 receptor in the processes involved in major depressive disorders, such as damaged monoaminergic neurotransmission [23,24], enhanced glutamatergic neurotransmission [25], neuroinflammatory response [26], and repressed neuroplasticity [24,27]. Additionally, our previous study demonstrated the augmentative effect of the P2X7 receptor on comorbid diabetic neuropathic pain and depression [28]. The close association between the P2X7 receptor and inflammation and the fact that IBS patients display both mucosal and neural inflammation motivate us to assume that, by inhibiting the P2X7 receptor, comorbid chronic visceral pain and depression could be alleviated.

Gallic acid (GA), which is found in a wide variety of fruits, nuts, and plants (e.g., rhubarb, eucalyptus, Cornus), is a polyphenol organic compound that is also known as 3,4,5-trihydroxy benzoic acid [29,30]. Its anti-inflammatory effects in various diseases have been demonstrated in many studies [31], for example, diabetes mellitus [32], psoriasis [33], gouty arthritis [34], paraquat-induced renal injury [35], etc. Gallic acid may prevent the production of inflammatory factors downstream of NF-κB, such as IL-1β, TNF-α, and thioredoxin-like protein-4B [36]. Moreover, gallic acid can also mitigate pro-inflammatory responses by reducing the secretion of pro-inflammatory mediators, e.g., NO, PGE2, IL-6, etc., in a dose-dependent manner [37]. In addition to the anti-inflammatory effect of gallic acid, it was found that gallic acid could cross through the liposome membrane to react with the 1,1-diphenyl-2-picryl-hydrazyl (DPPH) free radical and had an antioxidant effect in preventing the injury of oxidative stress in neurodegenerative diseases [38]. Furthermore, it was shown to have anti-depressant properties in chronic stress mice models [39], arsenic-induced brain injury rat models [40], and post-stroke depression rat models [41]. The anti-inflammatory and anti-depressive properties of gallic acid render it a possible candidate to treat comorbid visceral pain and depression.

In this study, we aimed to study the potential beneficial effects of gallic acid on comorbid visceral pain and depression, to determine whether gallic acid can alleviate the comorbidity by affecting the P2X7 receptors in the hippocampus, spinal cord, and dorsal root ganglion (DRG) and to investigate the possible mechanism.

## 2. Results

### 2.1. Molecular Docking of Gallic Acid to P2X7 Receptors

**Figure 1:**
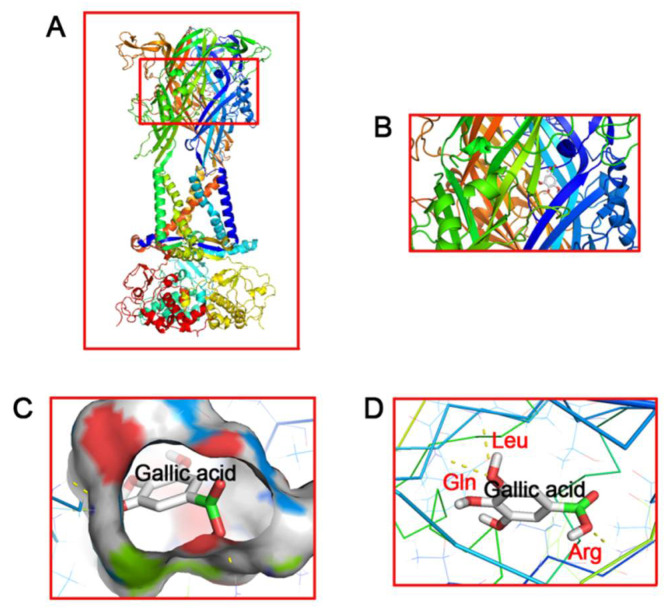
Molecular docking of gallic acid (GA) to the P2X7 receptor. The simulation modeling of GA docking to the P2X7 receptor was performed by a computer. The molecular docking prediction of GA to the P2X7 receptor was performed using AutoDock 4.2. The front view (**A**), top view (**B**) and enlarged views (**C**,**D**) indicate the perfect match for GA to interact with the P2X7 receptor.

The results of molecular docking show that gallic acid binds the P2X7 receptor at a binding pocket made up by P2X7 receptor B and C chains via hydrogen bonds. Figure 1 shows the binding patterns of gallic acid and the P2X7 receptor in different fields, where different colors represent different side chains. The results of molecular docking also show that the binding affinity of gallic acid to the P2X7 receptor is 6.4 (kcal/mol) (Table 1). The absolute value of binding affinity >6 kcal/mol being set as the standard, the binding affinity of gallic acid to the P2X7 receptor was considered good.

The predicted binding affinity is in kcal/mol (energy). * rmsd: RMSD values were calculated relative to the best mode and only used movable heavy atoms. Two variants of RMSD metrics are provided: rmsd (RMSD lower bound: matches each atom in one conformation with itself in the other conformation, ignoring any symmetry) and rmsd/ub (RMSD upper bound: rmsd/lb [c1,c2] = max [rmsd’{c1,c2}, rmsd’{c2,c1}]; and rmsd’ matches each atom in one conformation with the closest atom of the same element type in the other conformation), which differ in how the atoms are matched in the distance calculation. There was a strong reaction between ligand and protein, and the molecular docking of gallic acid to P2X7 was stable.

### 2.2. The Effect of Gallic Acid on Hyperalgesia Threshold of Rats with Comorbid Visceral Pain and Depression

A total of 139 male seven-day-old suckling rats were selected for CRD. After 14 days of CRD and normal feeding to adulthood (8 weeks), 51 male rats were fully consistent with visceral pain and depression in behavioral tests, and the modeling rate was about 36% (comorbidity/overall × 100%). All depressive behaviors were caused by natural visceral pain rather than through manual intervention.

**Figure 2:**
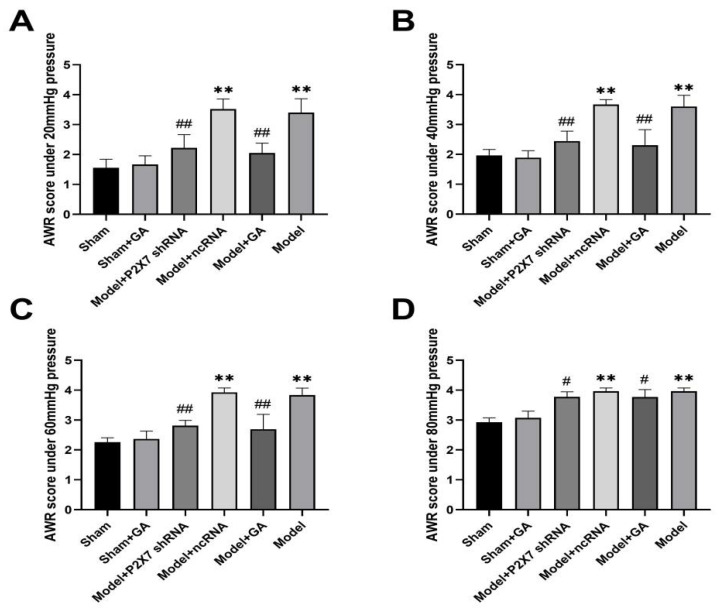
The chronic visceral hypersensitivity in rats was reflected in the AWR score in different groups under 20 mm/Hg (F(5,53) = 51.642, *p* < 0.001). (**A**) 40 mm/Hg (F(5,53) = 49.327, *p* < 0.001). (**B**) 60 mm/Hg (F (5,53) = 56.527, *p* < 0.001). (**C**) and 80 mm/Hg pressure (F(5,53)= 59.456, *p* < 0.001). (**D**) Values are means ± SEM. *p*-value was calculated by ANOVA. ** *p* < 0.01 vs. sham group; # *p* < 0.05 and ## *p* < 0.01 vs. model group.

The pain threshold was assessed using the AWR score. The score for each rat was the average score of two independent observers, each of whom conducted one observation every 30 min for three rounds. The scores of rats in the model group were significantly higher than those in the sham group under all pressures before treatment (*p* < 0.01), indicating that the visceral pain model was successfully established with a decreased pain threshold. The gallic acid intragastric administration (IA) protocol was performed once a day for 28 days, while the P2X7shRNA and ncRNA injection protocols were performed once a day for 7 days. The model group was intragastrically administered the same volume of solvent (DMSO + pure water) as the gallic acid preparation in the model + GA group. After 4 weeks of gallic acid IA on a daily basis or 1 week of P2X7shRNA intrathecal injection on a daily basis, the AWR score was identified as being significantly lower than that in the model group (*p* < 0.01) under the pressures of 20, 40, and 60 mm/Hg (Figure 2A–C). However, under the pressure of 80 mm/Hg, the remission effects of the gallic acid and P2X7shRNA were not evident (*p* < 0.05), as shown in Figure 2D. Under each pressure, the scores of the model and model + ncRNA groups remained significantly higher than those of the model + GA group and the model + P2X7shRNA group (*p* < 0.01), demonstrating that hyperalgesia was diminished after treatment with gallic acid or P2X7 shRNA. This indicated that both P2X7 knockdown and gallic acid could reduce the pain sensitivity of rats with visceral pain.

### 2.3. The Effect of Gallic Acid on Depression Levels of Rats with Comorbid Visceral Pain and Depression

**Figure 3:**
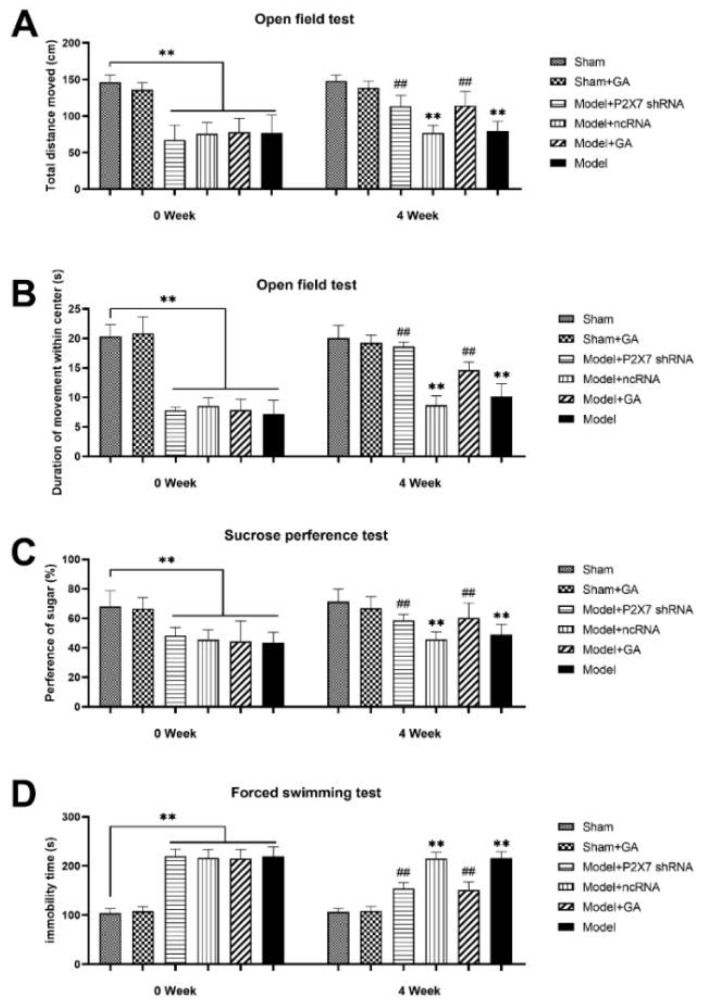
The depression levels of rats were reflected in the results of three independent behavioral tests (OFT, SCPT, and FST). Total moving distance (**A**) (F(5,53) = 42.329, *p* < 0.001) and duration of movement (**B**) (F(5,53) = 83.529, *p* < 0.001) within the center of the field before (0 week, 56 days age) and after (4 week) treatment in the OFT (5 min); preference of sugar (**C**) (F(5,53) = 15.273, *p* < 0.001) before and after treatment in the SCPT; IT (**D**) (F(5,53) = 140.105, *p* < 0.001) before and after treatment in the FST (5 min). The data in the first six columns of all bar charts are the data from before the treatment, and the data in the last six columns are the data from after the treatment. Every histogram bar includes the values from more than nine different samples. Values are means ± SEM. ** *p* < 0.01 vs. sham group; ## *p* < 0.01 vs. model group.

The weight of the selected rats was between 180 g and 250 g, and the rats were over 8 weeks old. The degree of depression was measured using three behavioral tests: the open field test (OFT), the sucrose preference test (SCPT), and the forced swimming test (FST). Before gallic acid IA, the results of the OFT demonstrated that the comorbidity model rats moved over a shorter distance (Figure 3A) (*p* < 0.01) and spent less time in the center of the field than those in the sham group (Figure 3B) (*p* < 0.01). After 4 weeks of gallic acid IA or 1 week of intrathecal injection of P2X7shRNA, the moving distances and time spent in the center of the field significantly increased for model + GA and model + P2X7shRNA rats (*p* < 0.01).

Combining the results of the SCPT and FST, the depression levels of rats were represented in the SCPT rates (sugar water consumption volume/total liquid consumption volume) and immobility time (IT). The results of the SCPT manifest that the comorbidity model rats had no preferences between sugar water and pure water; therefore, the SCPT rates were close to 50%. The comorbidity model rats presented shorter IT than sham rats, suggesting that the model group rats were more likely to be desperate in such oppressive environment (*p* < 0.01). However, rats treated by gallic acid or P2X7shRNA exhibited significantly increased SCPT rate values (Figure 3C) (*p* < 0.01) and reduced IT in the FST (Figure 3D) (*p* < 0.01) as compared with the model group.

The above results from the three behavior tests, i.e., OFT, SCPT, and FST, indicated that treatment with gallic acid or P2X7shRNA could relieve depression-like symptoms in the model rats.

### 2.4. Confirming Established Rat Visceral Pain Model by H&E Staining

**Figure 4:**
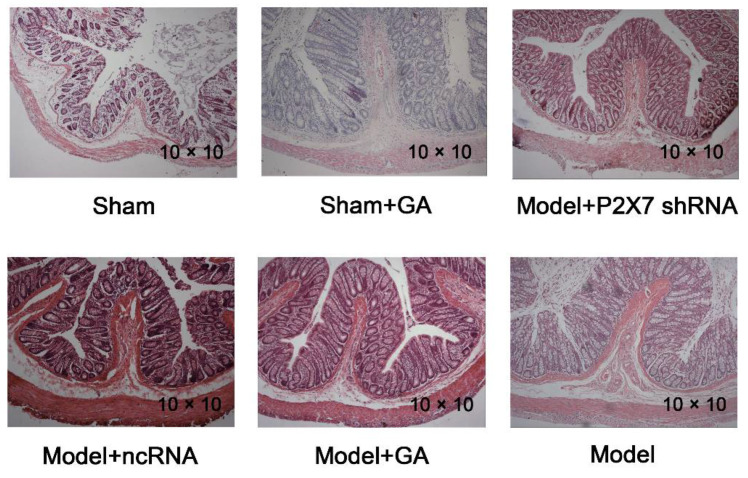
H&E staining of rat rectum tissues from each group under 100× field of view.

IBS is a type of functional bowel disorder (the intestinal expression of a gut–brain interaction disorder [42]) and is one of the most common diseases to involve visceral pain. However, CRD may damage the rectal structure in the process of modeling, resulting in organ damage, such as ulceration. Therefore, H&E staining was performed to exclude this. The results of H&E staining showed that the tissue structure of the colonic wall in each group was complete and uniform; the mucosal surface was smooth; and the intestinal glands in the lamina propria were regular. There were no obvious edemas in the surrounding stroma and no infiltrations of neutrophils, monocytes, or macrophages. It was shown that the modeling method did not lead to structural damages to the intestinal tracts of the rats, which was in accordance with IBS manifestation (Figure 4). By combining the behavioral test results of the rats (AWR score, OFT, SCPT, and FST), it can be inferred that the rat model of visceral pain with depression was successfully established in this study.

### 2.5. Effects of Gallic Acid on P2X7 Expression in the Hippocampi, Spinal Cords, and DRGs of Rats with Comorbid Visceral Pain and Depression

**Figure 5:**
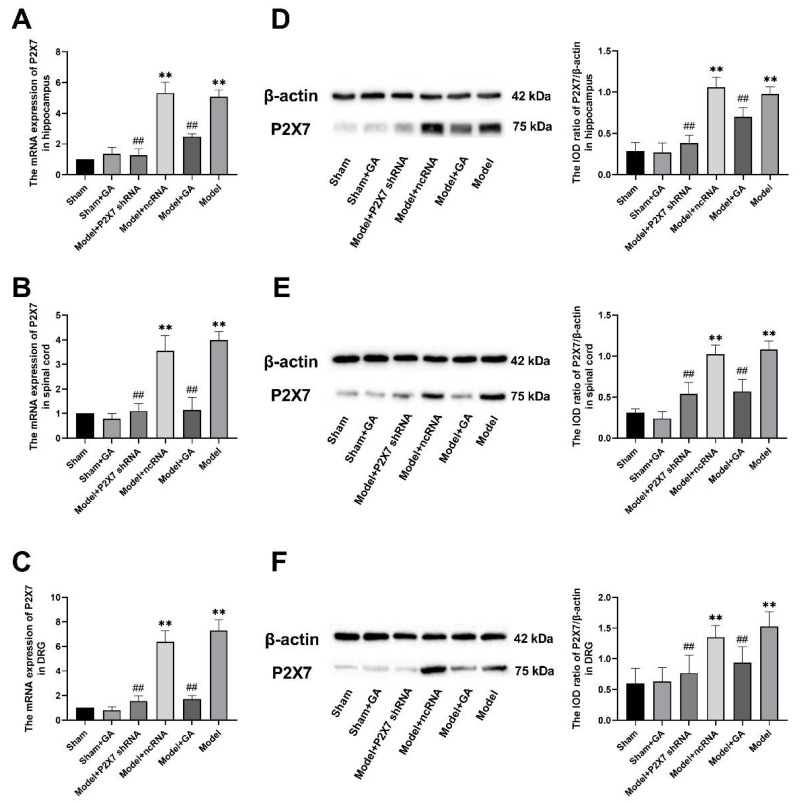
The expression of P2X7 receptor mRNA in the hippocampus was confirmed by qRT-PCR (F(5,30) = 131.475, *p* < 0.001) (**A**). The expression of P2X7 receptor mRNA in the spinal cord was confirmed by qRT-PCR (F(5,30) = 84.012, *p* < 0.001) (**B**). The expression of P2X7 receptor mRNA in the DRG was confirmed by qRT-PCR (F(5,30) = 31.043, *p* < 0.001) (**C**). β-Actin was used as the housekeeper gene in all qRT-PCRs. The relative expression of the P2X7 protein was detected by Western blotting in the hippocampus (F(5,30) = 68.997, *p* < 0.001) (**D**), spinal cord (F(5,30) = 62.397, *p* < 0.001) (**E**), and DRG (F(5,30) = 15.084, *p* < 0.001) (**F**). Values are means ± SEM. N = 6 per group. ** *p* < 0.01 vs. sham group; ## *p* < 0.01 vs. model group.

The mRNA levels and protein concentrations of the P2X7 receptor in the hippocampi, spinal cords, and DRGs of rats from each group were measured by qRT-PCR (Figure 5A–C) and Western blotting (Figure 5D–F). In all three tissue types, the expression levels of the P2X7 protein and mRNA in the model group were significantly higher than those in the sham groups (*p* < 0.01). By contrast, the mRNA and protein levels of the P2X7 receptor in the model + GA and model + P2X7shRNA groups were significantly lower than those of the model group (*p* < 0.01). Nevertheless, no significant differences were observed between the model + ncRNA group and the model group. These results demonstrated that gallic acid and P2X7shRNA could significantly reduce the expression of the P2X7 receptor in these tissues.

**Figure 6:**
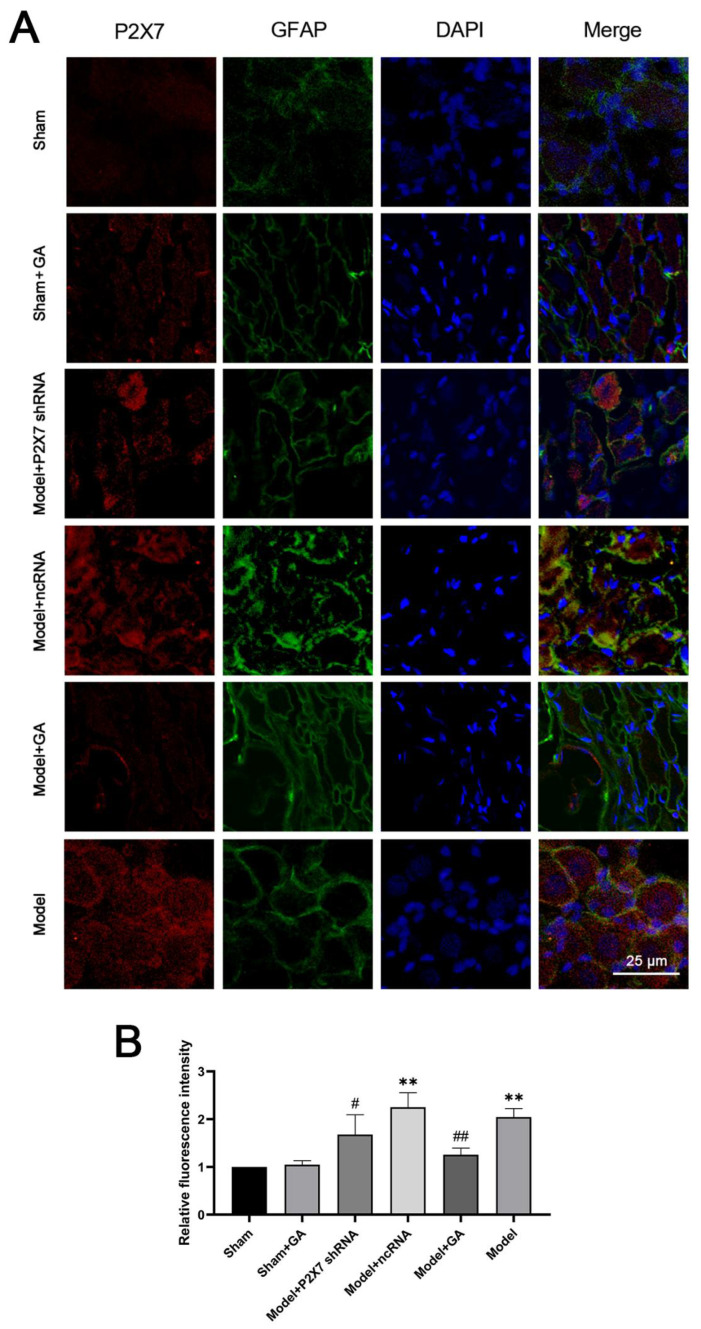
The effects of gallic acid on the co-expression of P2X7 and glial fibrillary acidic protein (GFAP) in the DRG. The blue signal indicates nuclei; the green signal indicates GFAP; and the red signal indicates the P2X7 receptor (**A**). The relative fluorescence intensity analysis (yellow) of the DRG (**B**) (F(5,30) = 31.126, *p* < 0.001). Values are means ± SEM. N = 6 per group. ** *p* < 0.01 vs. sham group; # *p* < 0.05 and ## *p* < 0.01 vs. model group.

**Figure 7:**
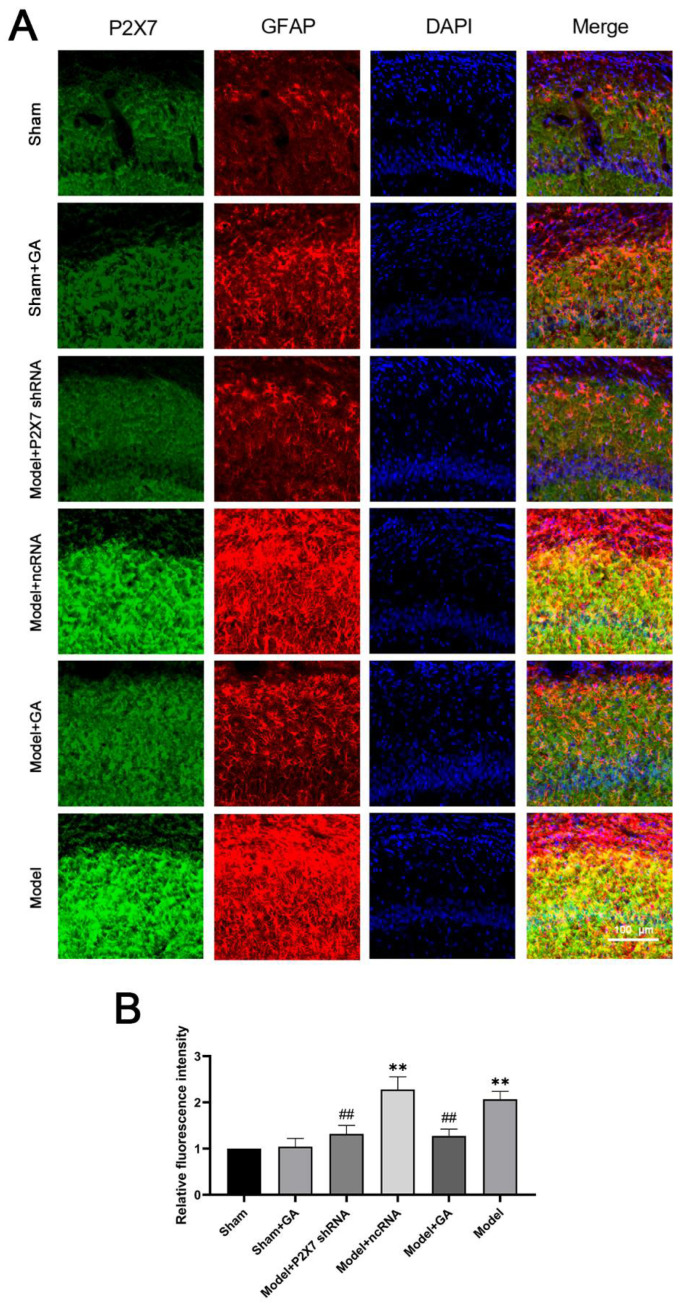
The effects of gallic acid on the co-expression of P2X7 and glial fibrillary acidic protein (GFAP) in the hippocampus CA1 area. The blue signal indicates nuclei; the green signal indicates the P2X7 receptor; and the red signal indicates GFAP (**A**). Relative fluorescence intensity analysis (yellow) of the hippocampus (**B**) (F(5,30) = 54.229, *p* < 0.001). Values are means ± SEM. N = 6 per group. ** *p* < 0.01 vs. sham group; ## *p* < 0.01 vs. model group.

The alterations in the expressions of the P2X7 receptor in the hippocampus and DRG were also confirmed in the immunofluorescence results. In the immunofluorescent image of the hippocampus (Figure 6A), green represents the P2X7 receptor, and red represents GFAP. However, in the DRG (Figure 7A), the colors are the opposite to facilitate differentiation. Despite the changes in the P2X7 receptor levels in both the DRG and hippocampus, the findings also show that, in the hippocampus, the levels and number of GFAP decreased in the model with gallic acid and the P2X7shRNA administration groups as compared with the model and ncRNA addition groups (*p* < 0.01). Additionally, the gallic acid and the P2X7shRNA administration groups demonstrated a low intensity of co-expressions of GFAP and P2X7 receptors, as was expected (Figure 6B and Figure 7B). These results suggested that gallic acid could reduce the co-expressions of GFAP and P2X7 receptors in hippocampus and DRG.

### 2.6. Effects of Gallic acid on ERK1/2 Phosphorylation in the Hippocampi, Spinal Cords, and DRGs of Rats with Comorbid Visceral Pain and Depression

**Figure 8:**
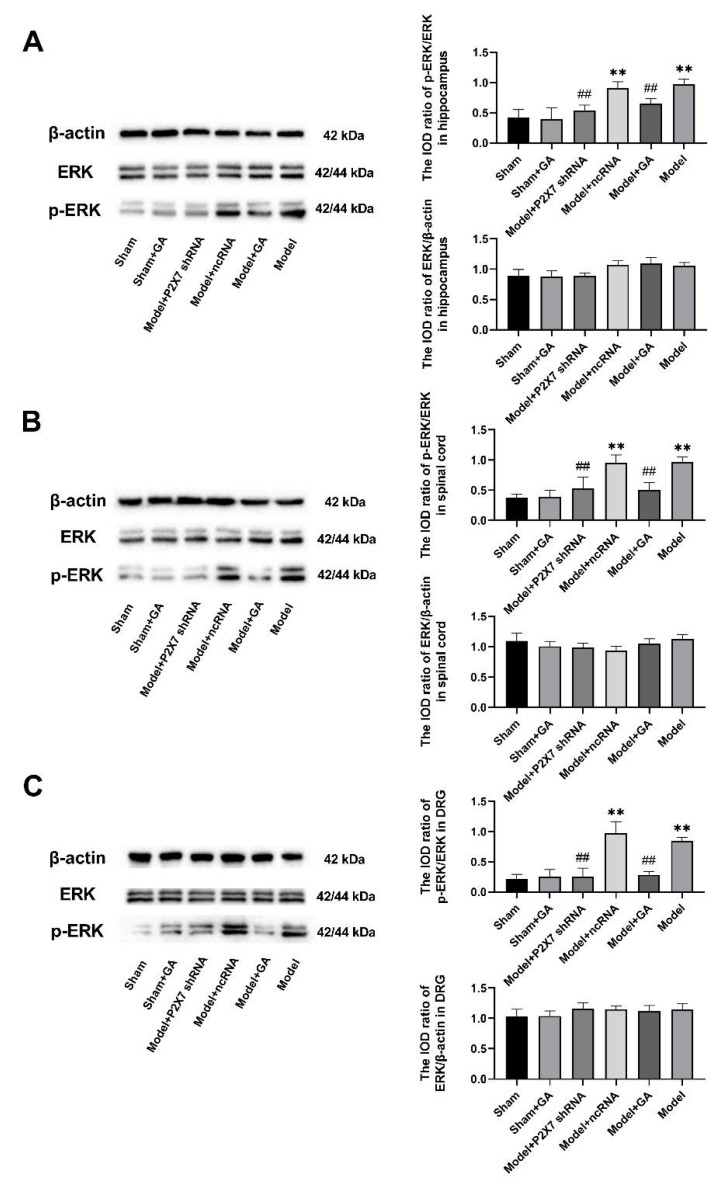
The effects of GA on ERK and phosphorylation of ERK1/2 in the hippocampus (**A**) (F(5,30) = 25.222, *p* < 0.001), spinal cord (**B**) (F(5,30) = 30.171, *p* < 0.001), and DRG (**C**) (F(5,30) = 55.567, *p* < 0.001) were determined by Western blotting. β-Actin was used as the housekeeper gene in all tissue types. Values are means ± SEM. N = 6 per group. ** *p* < 0.01 vs. sham group; ## *p* < 0.01 vs. model group.

The levels of ERK1/2 and Phospho-ERK1/2 (p-ERK1/2) in the hippocampus, spinal cord, and DRG were measured by Western blotting. The concentration of ERK1/2 in each tissue from different groups was basically identical, but the levels of p-ERK1/2 in the model and ncRNA groups were significantly higher than those in the sham group (*p* < 0.01). This suggests that the phosphorylated (activated) state of the ERK1/2 protein performed significantly in the course of the comorbidity. Meanwhile, the expressions of p-ERK1/2 in the model + GA and model + P2X7shRNA groups were greatly lower than that in the model group. Therefore, gallic acid and P2X7shRNA treatments in rats with visceral pain and depression largely reduced p-ERK1/2 expression (*p* < 0.01) (Figure 8). There were no significant differences in the expression level of p-ERK1/2 between the model + ncRNA group and the model group.

### 2.7. Effects of Gallic Acid on Serum IL-1β, IL-10, and TNF-α in Rats with Comorbid Visceral Pain and Depression

**Figure 9:**
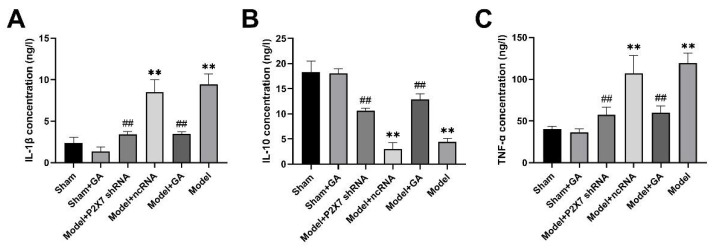
The effects of GA on the concentration of interleukin 1β (IL-1β) (**A**) (F(5,30) = 85.322, *p* < 0.001); interleukin 10 (IL-10) (**B**) (F(5,30) = 172.816, *p* < 0.001); and tumor necrosis factor α (TNF-α) (**C**) (F(5,30) = 56.655, *p* < 0.001) in the serum of rats in each group were assessed using ELISA. Values are means ± SEM. N = 6 per group. ** *p* < 0.01 vs. sham group; ## *p* < 0.01 vs. model group.

The concentrations of IL-1β, IL-10, and TNF-α in the serum of rats in each group were detected by ELISA. Both IL-β and TNF-α are pro-inflammatory factors. The results showed that IL-1β and TNF-α in the model group were significantly higher than those in the sham groups (*p* < 0.01), whereas gallic acid and P2X7shRNA significantly decreased IL-β and TNF-α levels as compared with the model groups (*p* < 0.01) (Figure 9A,B). As an anti-inflammatory factor, the concentration of IL-10 exhibited an opposite trend; it was significantly lower in the model group than in the sham group (*p* < 0.01), while gallic acid and P2X7shRNA significantly increased serum IL-10 levels in visceral pain and depression rats (*p* < 0.01) (Figure 9C). There were no significant differences between the model group and the model + ncRNA group in all experiments. Therefore, the data demonstrated that gallic acid and P2X7 shRNA had evident anti-inflammatory effects and attenuated the comorbidity.

### 2.8. Effects of Gallic Acid on mRNA Levels of IL-1β, IL-10, TNF-α, and BDNF in the Hippocampus of Rats with Comorbid Visceral Pain and Depression

**Figure 10:**
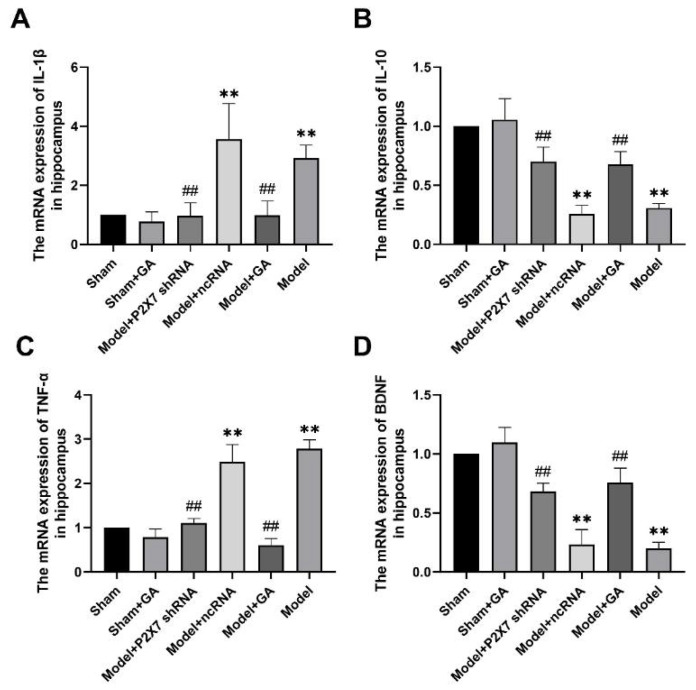
The effects of GA on the mRNA level of IL-1β (**A**) (F(5,30) = 24.350, *p* < 0.001); IL-10 (**B**) (F(5,30) = 62.094, *p* < 0.001), TNF-α (**C**) (F(5,30) = 123.893, *p* < 0.001); and brain-derived neurotrophic factor (BDNF) (**D**) (F(5,30) = 94.780, *p* < 0.001) in the hippocampus of rats in each group were assessed using qRT-PCR. β-Actin was used as the housekeeper gene in all qRT-PCR. Values are means ± SEM. N = 6 per group. ** *p* < 0.01 vs. sham group; ## *p* < 0.01 vs. model group.

The expressions of IL-1β, IL-10, TNF-α, and brain-derived neurotrophic factor (BDNF) at the mRNA level in the hippocampus of rats in each group were detected by qRT-PCR. The results showed that the expressions of both IL-1β and TNF-α in the model and model + ncRNA groups were significantly higher than those in the sham group (*p* < 0.01), while gallic acid and P2X7shRNA significantly decreased their expressions (Figure 10A,B). However, the expression trend of IL-10 and BDNF was opposite to that of IL-1β and TNF-α. Their expressions in both the model group and the ncRNA group were downregulated as compared with the sham group (Figure 10C,D). By contrast, gallic acid and P2X7shRNA were able to reverse the changes in IL-10 and BDNF. The data suggested that neuroinflammation was crucial in the development of the comorbidity, which could be significantly suppressed by gallic acid or P2X7 shRNA. The concentration change in BDNF was negatively related to the depressive level. Therefore, we affirmed that the expression of the P2X7 receptor was negatively associated with BDNF concentration, and gallic acid could alleviate depression via P2X7 receptor downregulation and BDNF elevation.

## 3. Discussion

The co-occurrence of pain and depression is commonly seen in clinics. It has been reported that the depletion of both serotonin and norepinephrine seen in depression patients interferes with the pain modulatory system [43]. Moreover, many patients with chronic pain are found to suffer from major depressive disorder (MDD) [43,44]. It is well substantiated that pain at baseline, pain severity, and chronicity are statistically related to MDD [45]. Comorbid pain and depression patients show both unsatisfactory pain and depression medication responses [46,47].

The incidence of visceral pain in patients suffering depression is very high. IBS is the most common disease associated with chronic visceral pain, and it is estimated that 20–60% of IBS patients also deal with depression [2]. In this study, a rat model of comorbid visceral pain and depression was used to conduct experiments. Rats were treated with a series of colorectal balloon distension (CRD) applications as neonates resulting in visceral pain that persisted into adulthood [48]. The IBS model was verified by both abnormally high AWR scores and structurally normal rectums in the H&E staining images of IBS rats. The co-existence of depression was also testified by the apathetic performances in the SCPT, OFT, and FST. It emerged that around 84.1% (visceral pain/overall × 100%) of SD rats (male) developed visceral pain after neonatal CRD, of which 43.6% (comorbidity/visceral pain × 100%) displayed both visceral pain and depression. The results of H&E staining showed that the tissue structure of the colonic wall in each group was complete and uniform, which demonstrated that the modeling method did not lead to structural damage to the intestinal tract of the rats. Thus, the hypersensitivity of the nervous system is crucial in visceral pain development in this study [49]. It has been reported that neonatal CRD contributes to the vulnerability of hippocampal microglia, which are more susceptible to being sensitized during adult CRD and to release a plethora of cytokines. This brings about a reduction in the hippocampal glucocorticoid receptor [50], consequentially augmenting corticotropin-releasing hormone (CRH), stimulating HPA, central neuronal sensitization, and spinal sensitization [51]. Thus, in this study, many rats suffering from visceral pain naturally exhibited depression-like behavior without additional stimulation. However, only few studies have established a model of comorbid visceral pain and depression as the investigation subject. Thus, our work provides a novel approach that can be used to understand the underlying molecular mechanisms involved in comorbid visceral pain and depression.

Gallic acid is a phenolic acid that is anti-inflammatory, antioxidant, and anti-depressive [37,38]. Previous findings show that the neuroprotective effect of gallic acid has been verified under many pathological conditions, such as neurotoxicity related to glutamate [52], cobalt chloride [53], arsenic [40], and aluminum chloride [54]; neurodegeneration induced by type-2 diabetes [55] and metabolic syndrome [56]; etc. Upon aflatoxin B1 neural toxification, the application of gallic acid displays neuroprotective properties via anti-inflammatory, antioxidant, and anti-apoptosis mechanisms [57]. Our laboratory has been investigating the pharmacological effects of gallic acid and proved that it can be neuroprotective against neuropathic pain via inhibiting the P2X7 receptor-mediated NF-κB/STAT signaling pathway [58]. In our study, we showed the combination of gallic acid and the P2X7 receptor by molecular docking analysis, which was also supported in a report by Yang Runan et al. [58]. This study is aimed at investigating the effect of gallic acid on the comorbid visceral pain and depression and determining whether gallic acid could affect the expression of the P2X7 receptor.

In this study, we found that the expressions of the P2X7 receptor in the hippocampi, spinal cords, and dorsal root ganglions (DRGs) of rats with visceral pain and depression increased significantly as compared with normal rats. The results show that the P2X7 receptor could modulate IBS, which is consistent with previous reports in our laboratory [15]. As a potent mediator of inflammation, the P2X7 receptor’s relationship with inflammatory markers has been well studied [59]. Resting cells originally possess the inactive precursor of casp-1-procaspase-1 (procasp-1). Once ATP binds to the P2X7 receptor, the activated P2X7 receptor conducts K^+^ efflux; then, procasp-1 is proteolytically activated into casp-1 in the “IL-1β inflammasome protein complex” [60]. Thereafter, casp-1 converts pro-IL-1β (inactive) into IL-1β (active), and IL-1β is released into the pericellular space [61]. Additionally, it was also demonstrated that the P2X7 receptor could modulate the release of IL-18 from monocytes [62]. The contact of IL-18 and the αβ heterodimeric receptor causes the synthesis of other cytokines, e.g., IL-6, IL-8, TNF-α, IL-1, and IFN-γ [63]. Our results support that IBS rats with depression exhibited higher P2X7 receptor expression in the spinal cord and dorsal root ganglia—the key central factors in the lower portion of the GIT sensory system [64]. Furthermore, the P2X7 receptor is vital for NLRP3 inflammasome activation, which was found to facilitate depressive behaviors [65]. In this study, we also witnessed significantly increased P2X7 receptor expression in the hippocampus of comorbidity model rats, suggesting that elevated hippocampal P2X7 receptor expression might induce depressive behaviors. Thus, our findings substantiated that increased P2X7 receptor expression in the hippocampus, spinal cord, and DRG may promote visceral pain and depression. In this research study, we also investigated the effects of gallic acid and P2X7 shRNA treatment on visceral-pain-associated depression. Our results indicate that gallic acid or P2X7 shRNA treatment can diminish spinal cord, DRG, and hippocampus P2X7 receptor expressions, further substantiating that gallic acid has a similar down-regulating effect on the P2X7 receptor expression as P2X7 shRNA. Animal behavioral tests suggested that a lowered pain threshold and elevated depression degree in models were restored to normal via gallic acid or P2X7 shRNA administration. Therefore, the generation of comorbid visceral pain and depression is associated with the elevation in P2X7 receptor expression, which gallic acid helps restore to normal. Furthermore, serum and hippocampal IL-1β and TNF-α exhibited a similar alteration trend among model rats as the P2X7 receptor, while serum and hippocampal IL-10 and hippocampal BDNF exhibited an inverse tendency. It has become common to treat depression according to the inflammatory mechanism and pro-inflammatory cytokines (such as IL-1 β, IL-6, and TNF-α). The decrease in the IL-10 factor caused by low-grade colonic mucositis is considered to play an important role in the pathophysiology of IBS [66]. The levels of pro-inflammatory cytokines are often considered to be screening biomarkers to predict whether the anti-depression treatment is effective or not [67], as it has been widely shown that neuroinflammation promotes MDD [68]. Moreover, BDNF, a neurotrophin that is of great significance for the neurons in key brain circuits associated with cognition and emotion, has anti-depressive activities [69,70]. In this study, the changes in IL-1β, IL-10, and TNF-α ulteriorly show that gallic acid improves the inflammatory environment both peripherally and centrally via the P2X7 receptor inhibition. Treatments with gallic acid and P2X7 shRNA successfully increased the BDNF level in the hippocampus of comorbid rats, indicating alleviated depression. The interaction of gallic acid and the P2X7 receptor has been testified by whole-cell patch-clamp tests [58], and the combination of gallic acid and the P2X7 receptor was found by molecular docking analysis in our research study. Incorporating all the results mentioned, we may draw the conclusion that the P2X7 receptor may be a common target for the comorbid visceral pain and depression, and the protective effect of gallic acid in comorbid rats is related to its anti-inflammatory property and increased BDNF in the hippocampus through P2X7 receptor inhibition.

To further investigate how gallic acid and P2X7 shRNA act on the mitigation of the comorbidities, the phosphorylation of ERK1/2 was explored by Western blotting. Mitogen-activated protein kinases (MAPKs) are a group of protein kinases that include p38, ERK1/2, and c-Jun N-terminal kinase (JNK) [71], among which the ERK subfamily has been found to be closely related to visceral pain and depression [28,72]. In a previous study, we found that P2X7 receptor shRNA reduced the increased levels of p-ERK1/2 in the DRGs, spinal cords, and hippocampi of rats with diabetic neuropathic pain and depression [28]. In our visceral pain and depression rat model, higher p-ERK1/2 levels (the activated form of ERK) were detected and could be blocked by treatment with either gallic acid or P2X7shRNA. Thus, it could be postulated that the mechanism of gallic acid relieving comorbid visceral pain and depression may be related to the inhibition of the ERK1/2 pathway.

One shortcoming of this study is that we did not investigate other possible pharmacological pathways of gallic acid. Gallic acid has multiple pharmacological effects, such as anti-inflammatory, antioxidant, and anti-apoptosis effects [73]. However, whether gallic acid acts on the P2X7 receptor alone or on other pathways remain to be defined in studies. Furthermore, the exact molecular mechanism still needs further research in the future for us to better understand the role of gallic acid in comorbid visceral pain and depression. The P2X7 receptor is thought to participate in both innate and adaptive immunity due to its wide presence on nearly all immune cells [74]. Many mechanisms of autoimmune diseases, neoplastic diseases, and degenerative diseases have been found to be associated with the P2X7 receptor [75,76,77]. Our study indicates that gallic acid can inhibit the P2X7 receptor, thus improving inflammatory conditions, which are shared by a plethora of autoimmune, neoplastic, and degenerative diseases. In order to determine whether gallic acid has therapeutic effects on those diseases, more studies that focus on its therapeutic range and its relationship with the P2X7 receptor are required.

## 4. Material and Methods

### 4.1. Molecular Docking

The P2X7.pdb file of the P2X7 receptor protein sequences was downloaded from http://www.rcsb.org/pdb/home/home.do (Accessed on 16 June 2021), and the gallic acid.sdf file was downloaded from https://pubchem.ncbi.nlm.nih.gov/. (Accessed on 19 June 2021). After pretreating with pyMOL software (The PyMOL Molecular Graphics System, Version 2.0 Schrödinger, LLC.) to remove small molecule ligands, dehydrate, and hydrogenate, autodock tools software (La Jolla, CA, USA) was applied for molecular docking based on python [28].

### 4.2. Animal and Treatment

SD rats were obtained from the Department of Animal Science, Jiangxi University of Traditional Chinese Medicine. The procedures were approved by the Animal Care and Use Committee at Nanchang University Medical School (SYKX2015-0001) and were performed according to the International Association for the Study of Pain’s ethical guidelines for pain research on animals. All the suckling rats along with their mother were housed in one plastic cage until day 21 after birth. At this point, the male rats were separated into other cages according to the experimental design, while the female rats were placed into other cages for mating. Female rats were excluded because the menstrual cycle would affect pain sensitivity [78].

Eight-day-old male suckling rats were selected to undergo neonatal colorectal dilation (CRD) [79]. In total, 93 suckling rats (n = 139) were used to establish the group of visceral pain combined with depression and 46 suckling rats to establish the sham operation group. During the period from the 8th day to the 21st day, neonatal CRD was regularly performed every day until the mother and baby were separated to establish the comorbidity (visceral pain + depression). The sham operation (sham) group was stroked on the anus with Vaseline at the same time.

**Figure 11:**
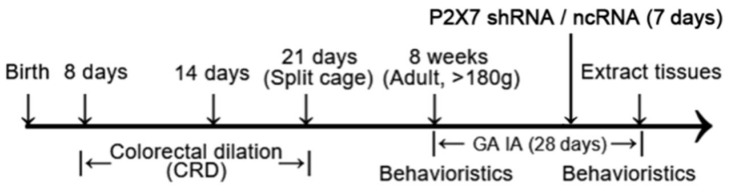
The flow chart showing the experimental design.

The hyperalgesia threshold and the level of depression of the rats were determined using behavioral tests (including OFT, FST, SCPT, and AWR scores as detailed below) when the rats reached 8 weeks. During this period, the rats did not undergo any depression treatment. After CRD treatment, rats that met the depressive requirements were named model and selected to be divided into four groups. The rats in the model + GA group were administered gallic acid (20 mg/kg) (Macklin Biochemical, Shanghai, China) intragastrically for 28 days; the rats in the model group were administered the same amount of solvent (DMSO + pure water) intragastrically for 28 days. The rats in the model + P2X7shRNA group were injected with P2X7shRNA intrathecally every day for 1 week; the rats in the model + ncRNA group were injected with non-code shRNA (ncRNA) intrathecally every day for 1 week. All rats were subjected to the same behavioral tests after treatment. The rats that underwent a sham operation during neonatal age were divided into two groups. The sham group continued without any treatment, and the sham + GA group was intragastrically administered the same concentration of gallic acid as mentioned above for 28 days. The experimental design is shown in Figure 11.

### 4.3. Drugs and Chemicals

The transfection complex, consisting of shRNA (P2X7shRNA or non-code shRNA) and transfection reagent at a ratio of 1:2 (μg/μL), was prepared using an Entranster™ in vivo transfection kit (Engreen Biosystem Company of Beijing), according to the manufacturer’s instructions. The complex was intrathecally injected into rats of the model + P2X7shRNA and model + ncRNA groups. The P2X7shRNA sequences are as follows:

*5′-CACCGTGCAGTGAATGAGTACTACGAATAGTACTCATTCACTGCAC-3′* and *3′-CACGTCACTTACTCATGATGCTTATCATGAGTAAGTGACGTGAAAA-5*′.

According to the literature [29] and the preliminary results from our laboratory, we decided to use gallic acid administrated intragastrically at a dosage of 20 mg/kg. The composition of gallic acid solution was 80 mg gallic acid + 160 μL DMSO + 9840 μL pure water (1:2:123).

### 4.4. Neonatal CRD

The 8-day-old male suckling rats were divided into 2 groups for different treatments. The model group was treated with neonatal CRD twice a day, from day 8 to day 21. This was performed with an angioplasty balloon and a sphygmomanometer. Firstly, the balloon smeared with Vaseline (Unilever, CT, USA) was inserted rectally into the descending colon of each rat. Then, a sphygmomanometer was applied to add 60 mmHg of pressure to the balloon for 1 min, after which the rat was released for a 30 min rest before the next round of CRD applications. In the sham group, the rats underwent 2 rounds of anal smearing with Vaseline on a daily basis from day 8 to day 21.

### 4.5. Adult CRD

After all the processed rats reached 8 weeks, adult CRD [79] was conducted to assess the pain threshold of each rat. Before CRD, each rat was intraperitoneally injected with 10% chloral hydrate for sedation. Then, a Vaseline-smeared balloon catheter was inserted rectally into the descending colon of each rat. After a 30 min interval for adaptation, the rat was placed on a flat table, and a sphygmomanometer was used to add 20, 40, 60, and 80 mm/Hg of pressure in a serial order. The pain threshold of each rat was determined by its behavior under each pressure level. According to the Abdominal Withdraw Reflex (AWR) score [79], head shaking represents the first degree of pain; slight abdominal muscle contraction without abdominal lifting represents the second degree of pain; abdominal lifting represents the third degree of pain; and an arching back represents the fourth and final degree of pain. By comparing the performance of the neonatal CRD group with the sham group, the rats with visceral pain that perceived more intense pain and thus had a lower pain threshold could be distinguished. These results were independently obtained and averaged by two observers without knowing the experimental details.

### 4.6. Sucrose Preference Test (SCPT)

After 24 h of liquid fasting, rats were kept in distinct cages containing 2 identical bottles holding 100 mL of sucrose water (10 g/L) and 100 mL of pure water. The sucrose preference (SCP) value of each rat was noted 1 h after bottle placement by measuring both pure water reduction (ΔP) and sucrose water consumption (ΔS) (SCP =ΔSΔS+ΔP×100%). The sucrose preference test (SCPT) is commonly used to evaluate anhedonia in animals, which refers to a reduced capacity to experience happiness [80]. By comparing the SCP values of the visceral pain group and the sham group, the rats with naturally derived comorbid visceral pain and depression were identified [28].

### 4.7. Open Field Test (OFT)

The open field test (OFT) imitates unsafe surroundings, evaluates animals’ autonomous behavior, and reveals how tense the animals are [80]. A 30 min dark adaptation was conducted before initiating the test. Then, one rat at a time was carefully placed in the center of a 40 cm × 50 cm × 60 cm open field. Two seconds after the Canon Powershot A610 camera (Canon Co. local distributer, Tehran, Iran) detected the animal, MATLAB (MathWorks Co., Natick, MA, USA) began to record the total traveled distance and the route for 5 min. The field was sanitized with 75% ethanol solution before the initiation of the following test [28].

### 4.8. Forced Swimming Test (FST)

The forced swimming test (FST) rat model was used to test the desperate depressive behavior [80]. An 80 cm high glass cylinder with an inner diameter of 40 cm containing 30 cm deep water at approximately 20 °C was utilized to hold one rat a time. Every rat was forced to swim for 5 min, and their motions were recorded. Thereafter, the immobility time (IT) of each test was calculated, which accounted for the total time the rat was immotile. By incorporating the performances of individual rats in all 3 behavioral tests, the ascertained visceral pain and depression rat models were separated [28].

### 4.9. Tissue Extraction

After 4 weeks of intragastric administration of gallic acid, rats were anesthetized with 10% chloral hydrate. DRGs, L4-L5 spinal cords, and whole brains were removed from a small number of rats and fixed in 4% PFA at 4 °C for 2 h. Subsequently, the tissues were dehydrated with 30% sucrose solution (in 4% PFA) at 4 °C for 24 h, during which the liquid was exchanged every 8 h. The other rats were beheaded for blood collection in a 20 mL EP tube. After centrifugation (3000 rpm/min) for 15 min, the upper serum was moved and stored at −80 °C. Then, their rectums, L4-L5 spinal cords, DRGs, and hippocampi were extracted and flushed with phosphate-buffered saline (PBS). The rectums were kept in EP tubes filled with PFA. Half of the remaining tissue was stored in EP tubes filled with RNA storage solution; the other half was left in PBS. All tissues were stored at −20 °C for further use.

### 4.10. Western Blotting

The stored spinal cords, DRGs, and hippocampi were put into different 2 mL homogenizers for the first round of homogenization in 98% RIPA lysis buffer, which is a mixture of 50 mM Tris-Cl (pH 8.0), 150 mM NaCl, 0.1% sodium dodecyl sulfate, 1% Nonidet P-40, 0.02% sodium deoxycholate, 100 mg/mL phenylmethylsulfonyl fluoride, and 1 mg/mL aprotinin. Next, 1% protease inhibitor and 1% phosphatase inhibitor were added into the homogenizers for the second round of grinding until no solid tissues were visible. Subsequently, all the homogenizers were left on ice for 30 min sedimentation before pouring the liquid into EP tubes and centrifuging at 12,000 rpm at 4 °C for 10 min. Thereafter, the supernatants were moved and mixed with 6 × loading buffer. The mixtures were heated in boiling water before cooling. They were then stored at −80 °C.

Extracted proteins were loaded on 12% sodium dodecyl sulfate–polyacrylamide gel for electrophoresis with a Bio-Rad system. The protein samples of the hippocampus and spinal cord in each well were 5–10 μg, while the protein samples of the DRG were 15–20 μg. Then, the protein bands were transferred onto polyvinylidene fluoride membranes (PVDF membranes), which were then blocked with 5% skim milk in 1×TBST (Cwbio, Beijing, China) for 2 h. After a gentle rinse, the membrane was immersed in the primary antibodies against β-actin (ZSGB-Bio, Beijing, China), the P2X7 receptor (Alomone, Jerusalem, Israel), extracellular signal–regulated kinases 1/2 (ERK 1/2) (Cell Signaling Technology, Danvers, MA, USA), and phosphorylated (p)-ERK1/2 (Cell Signaling Technology, Danvers, MA, USA) overnight at 4 °C. Thereafter, the membrane went through 3 rounds of 10 min washing with TBST before incubation in second antibodies horseradish peroxidase–conjugated secondary goat anti-rabbit IgG (ZSGB-Bio, Beijing, China) and goat anti-mouse IgG (ZSGB-Bio, Beijing, China) in blocking buffer on ice. Following another 3 rounds of 10 min washing, chemiluminescent solution (Advansta, Menlo Park, CA, USA) was dripped onto the membrane, which was then placed in the exposure machine (BIO-RAD, Hercules, CA, USA) for visualization of the protein bands. The grey density levels of the bands were measured with ImageJ.

### 4.11. Enzyme-Linked Immunosorbent Assay (ELISA)

The concentrations of IL-1β, TNF-α, and IL-10 in the collected serum samples were measured using relevant ELISA kits (Boster Biological Technology, WuHan, China). In brief, 50 μL of blank control buffer and 6 diluted standard solutions (720, 360, 180, 90, 45, and 22.5 μg/L) were added into the 96-well plate in triplicates. A mixture of 10 μL of serum samples of the different groups and 40 μL of standard solution were also added into the plate in triplicate. Then, the plate was sealed using a sealing membrane and incubated in a water bath for 30 min at 37 °C. Subsequently, the plate was washed for 5 times, and color developing agents were added into each well, followed by 10 min incubation at 37 °C in the dark. Finally, 50 μL of termination agent was added, and the absorbance of each well was measured using a microplate reader at 450 nm.

### 4.12. Quantitative Real-Time PCR (qRT-PCR)

The relevant materials were all ribozyme-free, and the homogenizers utilized went through acid soakage, diethyl pyrocarbonate soakage, autoclaving, and drying before use. The tissues (spinal cord, DRG, and hippocampus) were homogenized in RNA lysis solution, and RNA extraction was performed using a kit, according to the manufacturer’s instructions (Transgen, Beijing, China). Thereafter, 2 μL of the RNA from each sample was converted into complementary DNA using a RevertAid™ HMinus First Strand cDNA Synthesis Kit (TransGen, Beijing, China). The sequences of the primers used in the experiment were constructed using Primer Express 3.0 Software (Thermo Fisher Scientific, CA, USA). The sequences of the 6 pair primers were as follows.

β-actin forward *5′-TAAAGACCTCTATGCCAACA-3′* and reverse *3′-CACGATGGAGGGGCCGGACTCATC-5′.*

P2X7 forward *5′-GATGGATGGACCCACAAAGT-3′* and reverse *3′-GCTTCTTTCCCTTCCTCAGC-5′.*

IL-1β forward *5′-CCTATGTCTTGCCCGTGGAG-3′* and reverse *5′-CACACACTAGCAGGTCGTCA-3′.*

BDNF forward *5′-CCTCTGCTCTTTCTGCTGGA-3′* and reverse *5′-GCTGTGACCCACTCGCTAAT-3′*.

TNF-α forward *5′-CACGTCGTAGCAAACCACCAA-3′* and reverse *3′-GTTGGTTGTCTTTGAGATCCAT-5′.*

IL-10 forward *5′-CGGGAAGACAATAACTGCACCC-3′* and reverse *5′-CGGTTAGCAGTATGTTGTCCAGC-3′.*

The concentration of cDNA from each independent sample was 1000 ng/μL–1200 ng/μL. Quantitative real-time PCR was achieved using 0.1 mL 8-strip thin-wall PCR tubes with 300 ng of cDNA and 19.5 μL of master mix per well. β-Actin was used as housekeeper gene in all qRT-PCRs. Quantitative real-time PCR was conducted using StepOne (ABI, Thermo Fisher Scientific, CA, USA). The ΔΔCT method was used to quantify the expression of each gene, with CT as the threshold cycle. The relative levels of target genes normalized to the individual sample with the lowest CT are presented as 2^−ΔΔCT^. Each independent sample was tested three times to obtain the average value. The experimental results include six groups of different independent samples.

### 4.13. Hematoxylin–Eosin Staining (H&E Staining)

A total of 10 μm of the specimens was prepared with the rectum tissues stored in PFA. After splicing the wax blocks containing the rectums, the specimens were kept at 37 °C overnight. Then, the specimens were baked in an oven at 60 °C for 2–3 h before two rounds of dewaxing in xylene I and xylene II, respectively. Subsequently, the specimens went through a 5 min hydration in 100, 95, 75, and 50% ethanol in serial order, followed by 5 min of washing with PBS. Thereafter, the specimens were dyed with hematoxylin, which was followed by 5 min of rinsing under running water. Then, the specimens were differentiated with 1% hydrochloric acid alcohol before another a 1 h rinse under running water. Eventually, the specimens were colored for 15 s using eosin, dehydrated, and sealed using neutral resin.

### 4.14. Double-Label Immunofluorescence

DRGs, spinal cords, and hippocampi were sliced into specimens. First, these underwent 3 rounds of 5 min PBS washing and 4% paraformaldehyde fixation. Then, another 3 rounds of 5 min PBS washing were conducted before 1 h blocking with gout serum at 37 °C. Thereafter, the specimens were incubated in a mixed antibody solution containing both anti-P2X7 (1:100) (Alomone, Jerusalem, Israel) and anti-GFAP (1: 100) (Invitrogen, Carlsbad, CA, USA) at 4 °C overnight. Subsequently, the specimens were washed with PBS for 5, 10, and 15 min before being incubated in another mixed antibody solution for 60 min. For this, 2 protocols were utilized: the first was goat anti-rabbit tetramethylrhodamine (TRITC) 1:200 (Thermo Fisher Scientific, Carlsbad, CA, USA) and goat anti-mouse fluorescein isothiocyanate (FITC) 1:200 (Thermo Fisher Scientific, Carlsbad, CA, USA); the second was goat anti-mouse TRITC and goat anti-rabbit FITC 1:200. Then, the specimens were washed 3 times in PBS for 5 min and stained with 4′,6-diamidino-2-phenylindole (DAPI) for 5 min. The final products were sealed with anti-fluorescence attenuation agent.

### 4.15. Statistical Analysis

Statistical analyses were performed using SPSS26 software (IBM, New York, NY, USA) and GraphPad Prism (8.0.2, GraphPad software, San Diego, CA, USA). Data were analyzed by one-way analysis of variance (ANOVA) followed by the LSD post hoc test for multiple comparisons. The results are expressed as the mean ± standard error of the mean (SEM) and were considered statistically significant at *p* < 0.05.

## 5. Conclusions

In summary, our study suggests that the P2X7 receptor may be an effective target for both visceral pain and depression, and gallic acid could alleviate visceral pain and depressive behavior in rats by inhibiting the expression of the P2X7 receptor in the hippocampus, spinal cord, and DRG. The possible mechanism of gallic acid may be closely related to the inhibition of ERK1/2 phosphorylation and inflammatory cytokines. This study suggests that gallic acid is an effective drug to alleviate visceral pain combined with depression and is worthy of further clinical applications in the future.

## Figures and Tables

**Table 1:** Molecular docking score of P2X7 receptor docking and gallic acid.

Mode	Affinity	Dist from Best Mode	
	(kcal/mol)	rmsdl.b.	rmsdu.b.
1	−6.4	0.000	0.000
2	−6.3	16.856	18.085
3	−6.1	1.228	3.775
4	−5.7	17.977	19.492
5	−5.6	10.916	13.051
6	−5.6	10.910	13.236
7	−5.6	10.866	12.913
8	−5.6	9.819	11.322
9	−5.5	27.190	27.842

## Data Availability

The datasets generated during and/or analyzed during the current study are available from the corresponding author on reasonable request.
